# A novel method for identifying fine-scale bottom-use in a benthic-foraging pinniped

**DOI:** 10.1186/s40462-023-00386-1

**Published:** 2023-06-09

**Authors:** Nathan Angelakis, Simon D. Goldsworthy, Sean D. Connell, Leonardo M. Durante

**Affiliations:** 1 University of Adelaide, North Terrace, Adelaide, SA 5000 Australia; 2 South Australian Research and Development Institute (SARDI) (Aquatic Sciences), 2 Hamra Avenue, West Beach, SA 5024 Australia

**Keywords:** Bottom-use, Australian sea lion, Magnetometry, Dead-reckoning, Benthic forager, K-means clustering, Diving behaviour, Species management

## Abstract

**Background:**

For diving, marine predators, accelerometer and magnetometer data provides critical information on sub-surface foraging behaviours that cannot be identified from location or time-depth data. By measuring head movement and body orientation, accelerometers and magnetometers can help identify broad shifts in foraging movements, fine-scale habitat use and energy expenditure of terrestrial and marine species. Here, we use accelerometer and magnetometer data from tagged Australian sea lions and provide a new method to identify key benthic foraging areas. As Australian sea lions are listed as endangered by the IUCN and Australian legislation, identifying key areas for the species is vital to support targeted management of populations.

**Methods:**

Firstly, tri-axial magnetometer and accelerometer data from adult female Australian sea lions is used in conjunction with GPS and dive data to dead-reckon their three-dimensional foraging paths. We then isolate all benthic phases from their foraging trips and calculate a range of dive metrics to characterise their bottom usage. Finally, k-means cluster analysis is used to identify core benthic areas utilised by sea lions. Backwards stepwise regressions are then iteratively performed to identify the most parsimonious model for describing bottom usage and its included predictor variables.

**Results:**

Our results show distinct spatial partitioning in benthic habitat-use by Australian sea lions. This method has also identified individual differences in benthic habitat-use. Here, the application of high-resolution magnetometer/accelerometer data has helped reveal the tortuous foraging movements Australian sea lions use to exploit key benthic marine habitats and features.

**Conclusions:**

This study has illustrated how magnetometer and accelerometer data can provide a fine-scale description of the underwater movement of diving species, beyond GPS and depth data alone, For endangered species like Australian sea lions, management of populations must be spatially targeted. Here, this method demonstrates a fine-scale analysis of benthic habitat-use which can help identify key areas for both marine and terrestrial species. Future integration of this method with concurrent habitat and prey data would further augment its power as a tool for understanding the foraging behaviours of species.

**Supplementary Information:**

The online version contains supplementary material available at 10.1186/s40462-023-00386-1.

## Background

Identifying the core foraging areas used by a species is fundamental to understanding their ecology and essential for their effective conservation and management [1–3]. The accuracy and resolution of location data that informs these core areas is therefore essential to identify and understand important habitat and ecological requirements to maintain a species or population [4–6]. With advancements in tracking technology, obtaining movement data on marine species has improved markedly in recent decades, with smaller instrumentation and faster location acquisition times, giving higher spatial accuracy [4, 5, 7]. The use of these systems has since been crucial in elucidating at-sea movement, resulting in an improved understanding of the foraging ecology and management of a range of marine predators [8–10]. However, collecting movement data at high resolution can be challenging for diving species.

GPS and Argos locations can only be collected when an animal surfaces and correspondingly depends on reliable satellite constellations overhead [11–13]. For species that can spend extended periods underwater and have brief surface intervals, this can lead to large intermittent periods where no locational data are acquired [14–16]. Traditionally, movement-based models applied to diving species, linearly interpolate locations at regular time intervals between collected GPS or Argos locations [17–19]. However, by assuming straight-line travel between collected locations, linear models are limited in their ability to describe fine-scale, tortuous animal movement and can potentially miss important foraging activity. For the vast range of diving marine predators that forage at depth, such approaches can therefore lead to coarse descriptions of their sub-surface behaviour[15, 19–21].

One method that allows diving behaviour to be identified at high resolution, is the process of dead-reckoning, which uses accelerometry and magnetometry data to reconstruct underwater movement [14, 22, 23]. Dead-reckoning works by calculating an individual’s heading from magnetometer data (measuring relative direction from magnetic north) and speed and body orientation derived from accelerometer data. Magnetometers/accelerometers typically have low power consumption, so data can be sampled at very high resolutions for extended deployments on an animal. The benefits of using dead-reckoning over linear-interpolation models to describe movement of marine species are well documented. When compared with dead-reckoned tracks, linear movement models have shown significant mean position errors, large underestimations of total distances travelled by animals and inaccurate foraging area estimates [15, 22, 24]. Dead-reckoning has since been used to describe the movement of a range of terrestrial and marine species [25–28].

In this study we provide a novel method for identifying and mapping key foraging areas for an obligate benthic marine predator, the Australian sea lion (*Neophoca cinerea*) [29, 30]. Using k-means clustering, a method which partitions data into groups with similar features, we identify bottom-use from dead-reckoned foraging paths for three adult female Australian sea lions. Australian sea lions are currently listed as endangered under Australian legislation (*Environmental Protection and Biodiversity Conservation Act* 1999) and the International Union for the Conservation of Nature (IUCN) Redlist. Over the last 40 years the species has experienced a decline of 60% in total pup abundance [31]. Australian sea lions exhibit a high degree of foraging specialisation, both at individual and colony-specific levels and extreme site-fidelity to foraging areas, which they maintain throughout their lives [13, 32]. Management of Australian sea lions must therefore be targeted at a fine-scale level across the distribution of the species [33, 34]. This requires a detailed and high resolution understanding of how Australian sea lions are foraging in their local environment. Herein, we aim to 1) develop a novel method that allows high-resolution analysis of space-use for a benthic forager and 2), assess its ability to identify core benthic areas for Australian sea lions.

## Methods

### Study site

**Fig. 1:**
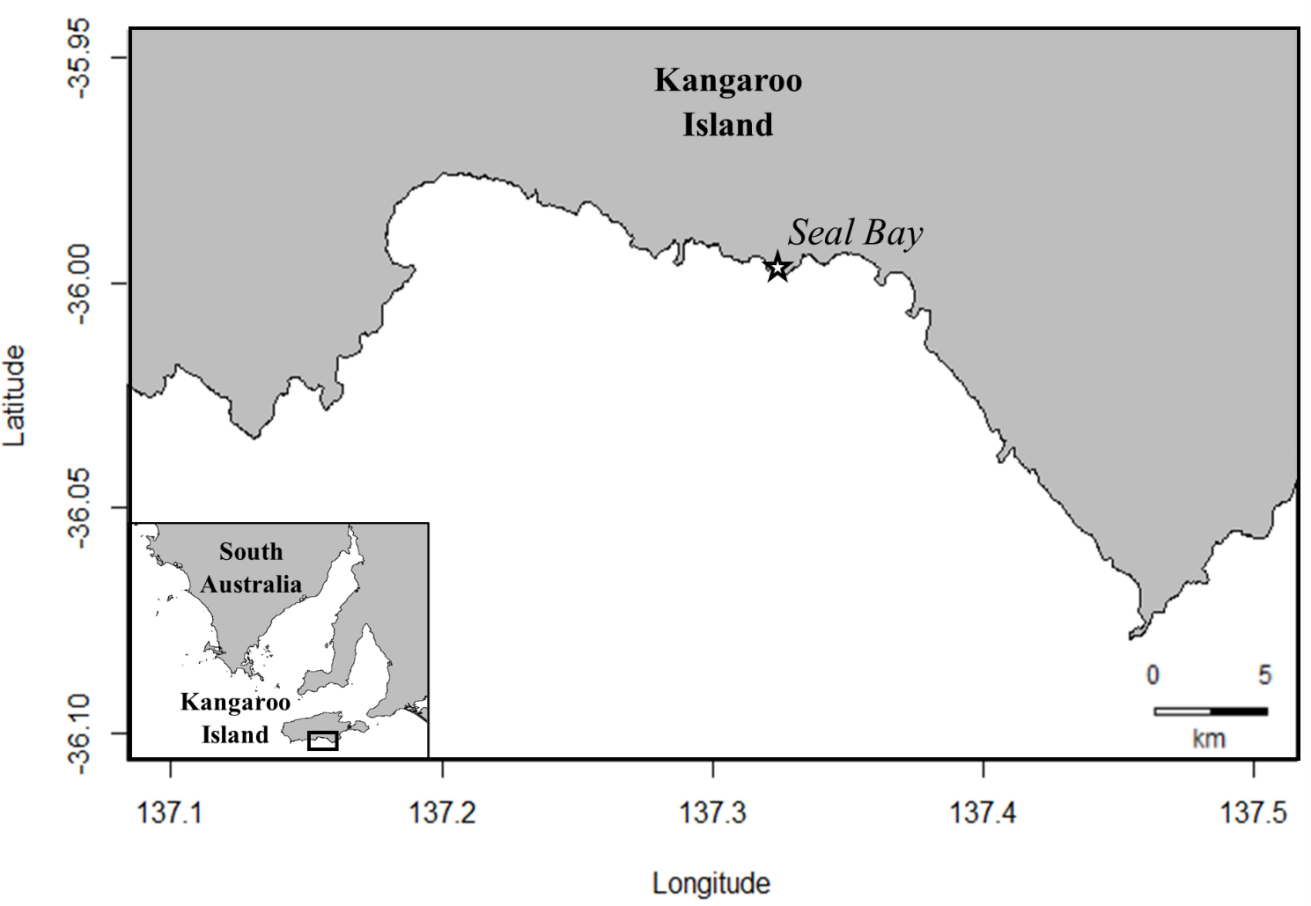
Location of the Australian sea lion colony at Seal Bay Conservation Park (35.994° S, 137.317° E), Kangaroo Island, South Australia, where microchipped animals were selected from

Data was collected from three adult female Australian sea lions (ages 14, 15 and 17) from Seal Bay Conservation Park (35.994° S, 137.317° E), Kangaroo Island, South Australia (Fig. 1) between October 2021 and February 2022. Age, body size, condition and reproductive information for each female (at deployment) is provided (Supplementary Table 1). Seal Bay is one of the largest Australian sea lion colonies (annual pup production = ~ 242) and a key monitoring site for the species with a microchipping program in place since 2002/03 that has microchipped over 80% of the population [35].

### Deployment of bio-logging devices

Sea lions were darted intramuscularly with Zoletil® (1.20–1.50 mg/kg, Virbac, Sydney, Australia) administered using remote syringe darts (Paxarms, 1.5- 3.0ml syringe body with 14-gauge 25 mm barbed needles, Paxarms New Zealand Ltd) fired from a dart gun (Paxarms MK24c Projector) to sedate them to a level that allowed safe application of an anaesthetic mask over the muzzle. Animals were then maintained under gas anaesthesia (~ 10–20 min) using Isofluorane® (5% induction, 0.5-3.0% maintenance), administered via a purpose-built gas anaesthetic machine with a Cyprane Tec III vapouriser (The Stinger™ Backpack anaesthetic machine; Advance Anaesthetic Specialists, NSW).

Bio-logging devices were all glued to pieces of neoprene (cut to the size of the device) that were then adhered to the dorsal pelage on the midline of sea lions (to minimise drag) using a two-part quick-setting epoxy glue (Selleys Araldite® 5 min Epoxy Adhesive). Accelerometer/magnetometers (Axy-5 XS, TechnoSmArt, 28 × 12 × 9 mm, 4gm) were positioned at the crown of the head (measuring head movement and orientation) and archival GPS/TDRs (time-depth recorders) (Mk10, Wildlife Computers, 100 × 55 × 28 mm, 214gm) were positioned at the base of the scapula. VHF transmitters (Sirtrack, 70 × 27 × 15 mm, 45gm) were used to aid in relocating animals for device recovery and were positioned above the tail. Devices with low profiles and mass were preferred to minimise the impact of drag on deployed animals. Instrumented sea lions were recaptured after a single foraging trip. Devices were removed by cutting them from their neoprene base to avoid damage to the pelage (neoprene bases are shed during the following moult).

### Data collection

Archival GPS loggers collected location data when animals surfaced by capturing a sub-second snapshot of radio signals from overhead satellite constellations. GPS loggers were programmed to collect a location at the minimum programmable Fastloc® rate, every two minutes, allowing a maximum of thirty Fastloc® measurements per hour. Integrated TDRs (time-depth recorders) measured depth every second.

Tri-axial accelerometers measured head movement (G-force) across surge (anterior-posterior), sway (lateral) and heave (dorsal-ventral) axes at 25 Hz and 8-bit resolution (maximum and minimum acceleration value ± 4G). Integrated magnetometers measured the earth’s magnetic field intensity in microteslas (µT) across roll (longitudinal, north), pitch (transverse, east) and yaw (vertical, down) axes at 2 Hz. Tri-axial accelerometers/magnetometers were calibrated prior to deployment on each individual by physical rotating the device across all of its three accelerometer and magnetometer axes. The minimum and maximum values collected across the three accelerometer and magnetometer axes during calibration were then used to standardise the data prior to processing.

### Location and depth processing

Locations from archival GPS loggers were obtained using Fastloc® GPS. Locations were acquired by converting the distances measured from the satellite ephemerides into position fixes. Locations derived from four or fewer satellites were discarded. Erroneous locations (those that represented unrealistic swimming speeds for sea lions) were removed via a speed threshold (speeds above 6 ms^− 1^ were omitted) [36] in *R* using the *trip* package [37]. Depth data provided by Mk-10 TDRs was zero-offset corrected to account for drifts in the TDR pressure transducer over the duration of the foraging trip.

### Dead-reckoning

Tri-axial accelerometer/magnetometer data was used to dead-reckon three-dimensional sub-surface movements, during times when GPS locations were not acquired. Depth data provided by TDRs were time-matched and amalgamated with tri-axial accelerometer/magnetometer data. Accelerometer/magnetometer data was standardised and calibrated for each individual by calculating slope and intercept vales via a linear model using the maximum and minimum values measured across each of their three axes during calibration. Foraging paths were estimated via dead-reckoning in the *TrackReconstruction* package [38]. Magnetic declination/inclination values (7.698, -68.012, World Magnetic Calculator) for the study area were provided. A high-pass filter with a running mean length of three seconds was used to isolate ‘dynamic’ acceleration (representing three-dimensional acceleration in head movement) from ‘static’ (gravity-based acceleration) [39, 40]. All speed-filtered longitudinal/latitudinal location data (in decimal degrees) was converted into radians to calculate consecutive bearing and distances between locations. Dead-reckoned calculations were then georeferenced by coercing the dead-reckoned ‘pseudo-track’ through all the consecutive GPS locations to provide an estimated reconstruction of three-dimensional movement. Data collected prior to the start and following the end of a foraging trip was omitted from analysis.

### Cluster analysis of core-bottom use

Dead-reckoned reconstructed paths and concurrent TDR (dive) data were then used to identify bottom-use by Australian sea lions using k-means clustering. Firstly, dive records for each individual were analysed using a unimodal model with a cubic regression spline in *R* using the *diveMove* package [41]. This model allows individual descent, ascent, bottom and surface phases for each dive across a TDR record to be identified. A 20-second window was used to smooth dive data across the TDR record. Individual dives for the duration of the foraging trip could then be identified. Their bottom phases, defined as when an animal is at > 80% of its maximum dive depth [13, 29], were isolated from the TDR record. A range of metrics to assess bottom-use/foraging effort (bottom time, time at depth, bottom distance, depth variability, bottom speed, bottom sinuosity, colony distance) were then calculated per dive, from the combined TDR and georeferenced, dead-reckoned data (Table 1). Australian sea lions are obligate benthic predators, which maximise bottom time [29, 30], with foraging restricted to shelf-waters [13, 32, 34]. Australian sea lions also exhibit strong individual/colony-specific specialisation and extreme fidelity to foraging areas, which they maintain throughout their lives [13, 32, 34]. These vectors were hence chosen to characterise the bottom-use of a philopatric, central-place forager, that works near its physiological limit to exploit the benthos [29, 30]. Co-linearity of these vectors was assessed via the Pearson correlation coefficient, those that showed high correlation (>0.750) were hence omitted from analyses.

K-means clustering was performed in base *R* using the methods described in Hartigan and Wong (1979) [42]. K-means clustering is a statistical method that partitions data into groups that share similar features. Using an unsupervised learning model, k-means clustering aims to minimise the within-cluster sum of squares and maximise the between-cluster sum of squares [42]. We used the k-means clustering algorithm to partition all bottom phases for each individual into groups based on the centres of a suite of predictor variables (Table 1). This provides a way of assessing bottom-use/foraging effort by Australian sea lions at the dive level. Data for each predictor variable was standardised by calculating z-scores for each observation, prior to application of the clustering algorithm. The optimal number of clusters for each individual was determined by analysis of average silhouette width plots and scores (indicating goodness of clustering between − 1 and 1, low-high) [43–45].

### Statistical analysis

**Fig. 2:**
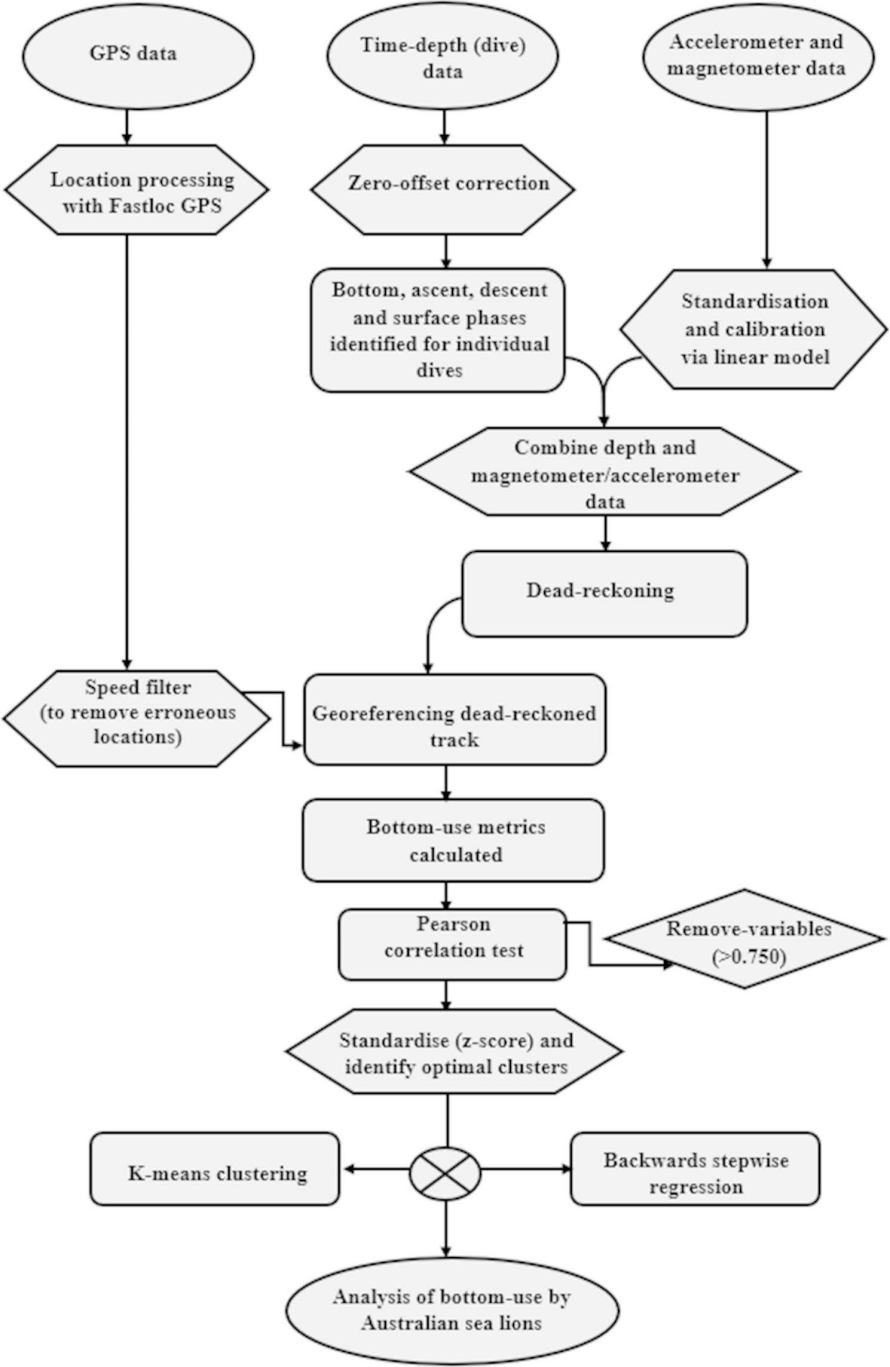
Flowchart of methodology used to analyse bottom-use in Australian sea lions. Shapes indicate different operational steps, representing start and end data objects (ovals), data preparation (hexagons), data processing (rectangles), methodological decisions (diamonds)

For each individual, backwards stepwise regressions were iteratively performed on the suite of predictor variables. This was done to confirm the selection of predictor variables to be included in the final k-means clustering algorithm. Variables with a p-value >0.05 were thus iteratively removed from the model, until the most parsimonious model was identified. Stepwise regression also allowed the significance of each term in the model to be identified. A flowchart of the methodology used in this study highlighting all key analytical steps is provided (Fig. 2).

**Table 1:** Dive predictor variables selected for analysing bottom-use via k-means clustering for three adult female Australian sea lions. ‘Bottom’ is defined as periods when animal is at >80% of maximum depth

Cluster predictor variables	Description of variable
**Bottom time**	Total bottom time (s)
**Time at depth**	Time at depth index. ∑ =total bottom time (s) / maximum dive depth (m)
**Bottom distance**	Total distance travelled at bottom (m)
**Depth variability**	Depth variability index. ∑ =standard deviation of bottom depth / mean bottom depth (m)
**Bottom speed**	Mean horizontal bottom speed (m/s)
**Bottom sinuosity**	Mean bottom sinuosity index. ∑ =distance travelled / straight-line distance (from start to end point of bottom phase)
**Colony distance**	Mean straight-line distance from colony (km)

## Results

### Location and dive data

A total of 931 at-sea Fastloc® GPS locations were available for analysis from three adult female Australian sea lions (Table 2). Foraging trip durations ranged from 47.38 to 85.30 h. Fastloc® GPS data from these three females hence provided on average, a reliable location approximately every 12 min. A total of 1,709 bottom phases were available for analysis of bottom-use by Australian sea lions. Mean bottom times ranged from 1 m:27 ss- 1 m:45 ss and mean dive times from 2 m:51 ss- 3 m:05 ss. Mean bottom depths ranged from 37.07 to 71.77 m and maximum bottom depths from 77.00 to 114.50 m (Table 2).

**Table 2:** Summary of location and dive data collected from three adult female Australian sea lions from Seal Bay Conservation Park (mean ±1 standard deviation)

ID	Number of locations	Trip duration (hh:mm)	Location frequency (no. h^− 1^)	Number of bottom phases	Mean bottom time (m:ss)	Mean dive time (m:ss)	Mean bottom depth (m)	Maximum depth (m)
**SB1**	253	47:23	5.33	520	1:45 ± 1:00	3:04 ± 1:17	37.07 ± 17.89	77.00
**SB2**	343	85:18	4.02	675	1:27 ± 0:48	2:51 ± 1:40	71.77 ± 31.15	114.50
**SB3**	335	56:44	5.90	514	1:30 ± 0:46	3:05 ± 1:07	47.30 ± 17.74	82.00

### Cluster analysis

For the three females presented in this study, analysis of average silhouette width plots and scores identified two distinguished clusters to group bottom-use into (Supplementary Fig. 1). Dives partitioned into the first cluster constitute a group with a lower mean bottom time, mean time at depth index, mean bottom distance and mean depth variability index. The second cluster represents a group with a greater mean bottom time, mean time at depth index, mean bottom distance and mean depth variability index. For SB1, dives grouped in the second cluster also share a higher mean bottom sinuosity than those in the first (Table 3). K-means clustering identified 45.58% and 54.42% of bottom phases for SB1 as cluster 1 and 2 respectively, 63.26% and 36.74% for SB2 and 52.72% and 47.28% for SB3 (Table 3).

Backwards stepwise regression determined models with four to five terms as the most parsimonious for applying the k-means clustering algorithm. Bottom speed and colony distance were iteratively removed from models for each individual (where p-value >0.05). Bottom sinuosity was iteratively removed from models for SB2 and SB3 (Table 3). For the clusters identified for SB1, bottom time and time at depth show the strongest statistical significance. For SB2, bottom time and bottom distance show the highest statistical significance and for SB3, bottom time and depth variability show the greatest statistical significance in the identified clusters (Table 3).

**Table 3:** Standardised centroid means for predictor variables from k-means clustering for three adult female Australian sea lions. Where *n* is the number of bottom phases for each cluster, adjusted R^2^ values are provided for each individual and p-values for each term included in the final model

ID	Cluster ID	*n*	Adjusted R^2^	Bottom time	Time at depth	Bottom distance	Depth variability	Bottom sinuosity
**SB1**	*1*	237		-0.710	-0.747	-0.487	-0.476	-0.150
	*2*	283	0.652	0.594	0.625	0.408	0.398	0.126
	***p***			**< 2e** ^**− 16**^ ***	**< 2e** ^**− 16**^ ***	**3.74e** ^**− 7**^ ***	**3.81e** ^**− 13**^ ***	0.00964 ***
**SB2**	*1*	427		-0.535	-0.479	-0.555	-0.079	-
	*2*	248	0.630	0.934	0.835	0.969	0.138	-
	***p***			**< 2.34e** ^**− 10**^ ***	**< 1.35e** ^**− 8**^ ***	**< 2e** ^**− 16**^ ***	0.0005 ***	-
**SB3**	*1*	271		-0.710	-0.747	-0.487	-0.476	-
	*2*	243	0.570	0.594	0.625	0.408	0.398	-
	***p***			**5.75e-** ^**12**^ ***	0.0002 ***	**8.76e** ^**− 12**^ ***	**< 2e** ^**− 16**^ ***	-

### Identifying core-bottom areas

**Fig. 3:**
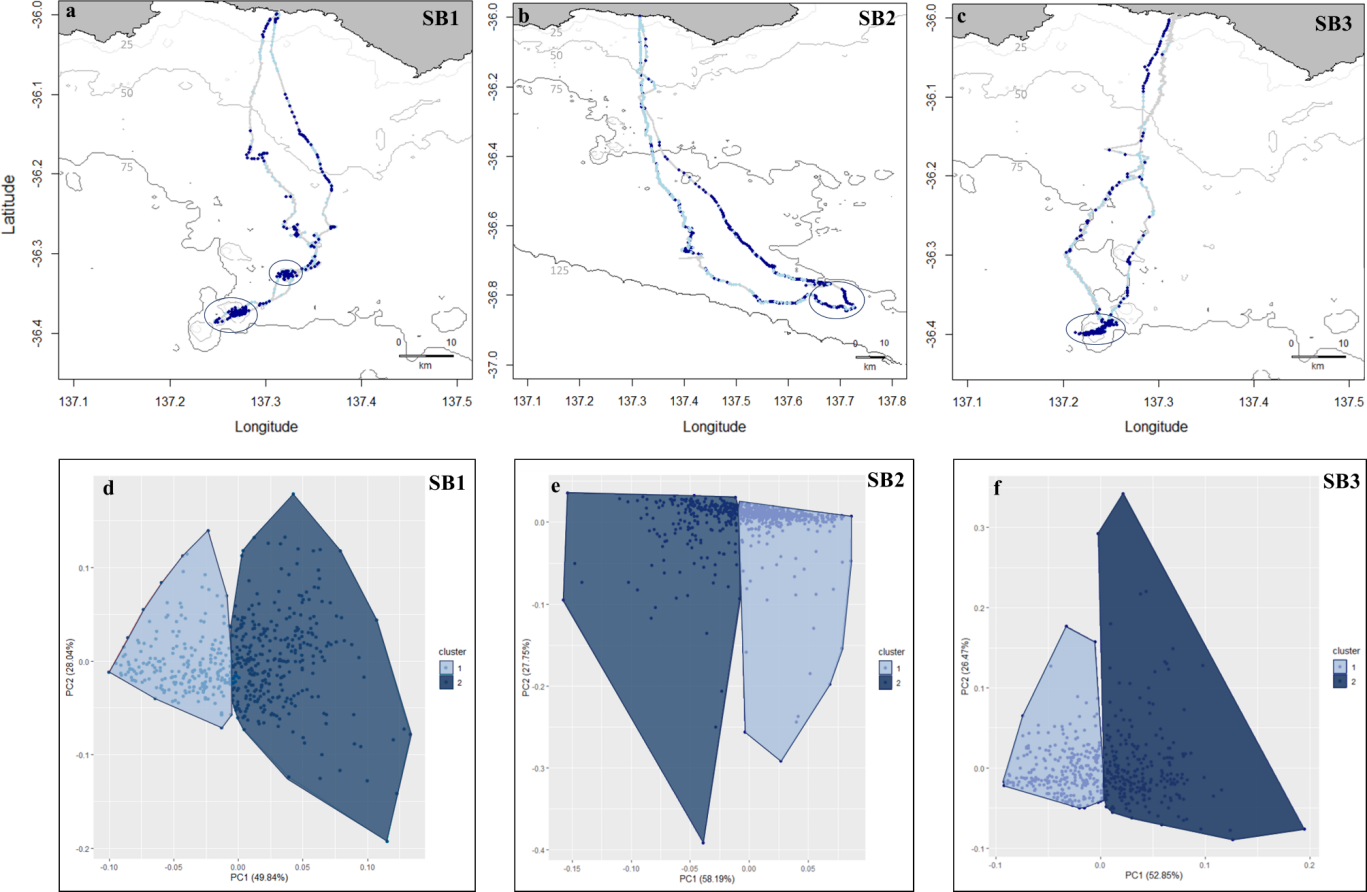
Core bottom-use areas identified by k-means clustering of dead-reckoned foraging paths for adult female Australian sea lions from Seal Bay, showing cluster 1 dives (lower foraging-effort) in light blue and cluster 2 dives (higher foraging-effort) in dark blue for individuals SB1 (a), SB2 (b) and SB3 (c). Aggregations of cluster 2 dives are circled in dark blue. Isobaths represent depth contours at 25, 50, 75 and 125 m (light to dark grey). K-means cluster plots, highlighting partitioning of bottom-use and principal components, are shown for individuals SB1 (d), SB2 (e) and SB3 (f)

K-means clustering of dead-reckoned paths for three adult female Australian sea lions shows spatial variation in partitioning of bottom-use (Fig. 3). For SB1 and SB3 aggregations of cluster 2 dives (higher foraging-effort dives) are evident towards the terminal end of their foraging trips. Smaller aggregations of cluster 2 dives are also shown for both SB1 and SB3 on their outward and colony-bound journeys (Fig. 3). K-means clustering of bottom-use for SB3 shows that majority of its cluster 2 dives are widely distributed across its colony-bound journey (Fig. 3). For individuals SB1 and SB3, k-means clustering has identified cluster 2 dives to be mainly distributed between 50 and 75 m, whereas for individual SB2 cluster 2 dives are mainly distributed between 75 and 125 m (Fig. 3).

**Fig. 4:**
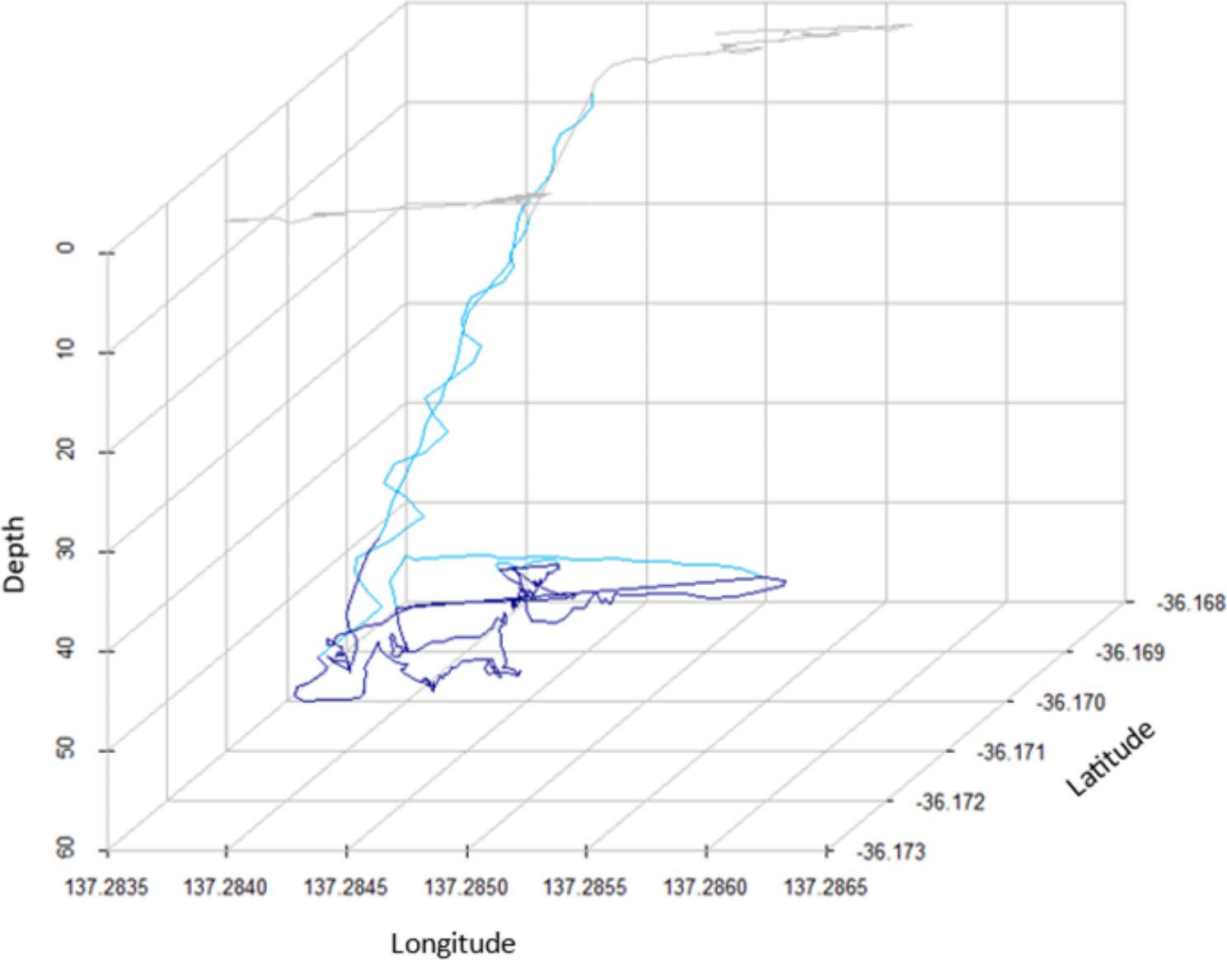
Three-dimensional dive profile from a dead-reckoned foraging path of an adult female Australian sea lion (SB1) from Seal Bay, highlighting fine-scale use of a core bottom area. Individual dive stages are highlighted for surface intervals (light grey), ascents/descents (light blue) and bottom phases (dark blue) for one foraging dive

**Fig. 5:**
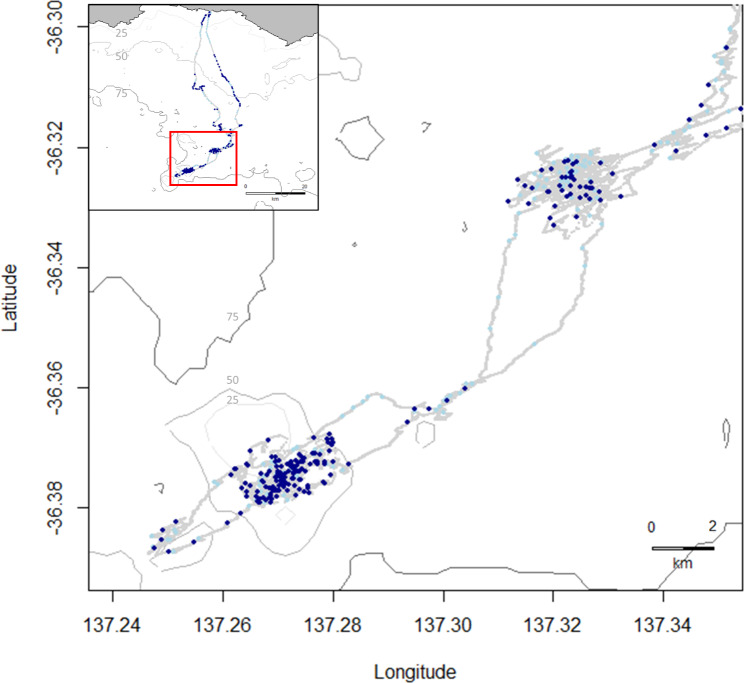
Dead-reckoned foraging path of an adult female Australian sea lion (SB1) from Seal Bay, highlighting tortuous, fine-scale bottom-use. Cluster 1 dives (lower foraging-effort) are highlighted in light blue and cluster 2 dives (higher foraging-effort) in dark blue. Isobaths represent depth contours at 25, 50 and 75 m (light to dark grey)

## Discussion

Analysis of dead-reckoned dive and location data from Australian sea lions has highlighted spatial partitioning in an individual’s benthic habitat-use (Fig. 3). In this study, k-means clustering has identified two major modes of bottom-use in adult female Australian sea lions. Those bottom phases grouped into cluster 2 represent dives where an individual has spent longer on the bottom, covered greater bottom distances, spent greater time at depth and targeted bottom habitats with greater rugosity (Table 3). Spatial analysis of sea lion bottom-use has identified aggregations of dives with greater bottom times, bottom distances and time spent at depth across habitats with more variable bathymetry (Fig. 3). These likely highlight key areas of activity where these sea lions are devoting greater foraging effort towards.

Australian sea lions focus foraging on maximising bottom time [29, 30], operating at high metabolic rates [29, 46, 47], to exploit bathymetric features that are predictable aggregation sites for benthic prey [48]. Cluster analysis of bottom-use in this study suggests that bottom time is a strong predictor for identifying core foraging areas for Australian sea lions (Table 3). For individuals SB1 and SB3, cluster analysis of bottom-use has highlighted key foraging areas over shallower bathymetric features at the terminal ends of their foraging trips (Fig. 3). Contrastingly, partitioning of key benthic-use for SB2 is distributed over deeper shelf waters, across a broader spatial scale. Foraging in Australian sea lions exhibits strong individual and colony-specific specialisation and extreme fidelity to foraging areas, which they maintain throughout their lives [13, 32]. Employing accelerometer/magnetometer data to analyse benthic habitat-use could therefore be a helpful tool in highlighting these individual differences in foraging strategies and behaviour. While previous use of magnetometer/accelerometer data to identify shifts in animal movement is rich [26–28], their application for identifying core-use areas/habitat is under-utilised. Along with the method presented here, studies that have used dead-reckoning to identify fine-scale habitat use for example in European badgers (*Meles meles*) [23] and Eurasian beavers (*Castor fibre*) [49] highlight key areas for future development.

Data collected from three adult female Australian sea lions highlights the advantages of incorporating dead-reckoning to identify the at-sea movements of a marine predator. While collected GPS data provided a reliable location approximately every 12 min (Table 2), the integration of accelerometer/magnetometer data has allowed collection of movement data at sub-second resolutions. This has allowed highly tortuous three-dimensional movements to be identified (Fig. 4), that otherwise would not be well detected by GPS alone or by linear movement models [20, 21]. In this study, dead-reckoning has illustrated the fine-scale targeting of benthic features in the marine environment by Australian sea lions (Figs. 3 and 5), highlighting differences in an animal’s space-use in relation to bathymetry (Fig. 5).

This method has allowed fine-scale analysis of bottom use of Australian sea lions at the dive level. A high-resolution understanding of space and habitat use is pertinent to Australian sea lions, particularly females, who show extreme philopatry [13, 32], where fine-scale species management is required [34]. Previous studies modelling core foraging areas for Australian sea lions have been fundamental in assessing, for example, interactions with fisheries [34, 50]. While these have been crucial for informing management of Australian sea lions on a state-wide scale [8, 31, 34, 50], the method presented in this study compliments past research, providing a fine-scale analysis that can be adapted to the colony/population level. This can help identify core foraging areas/habitat that may be missed by models that describe Australian sea lion distribution across a broader spatial scale.

While dead-reckoning can provide a high-resolution reconstruction of the sub-surface movements of diving species, the accuracy of the process is reliant on quality and temporally regular location data [15, 22, 24]. Due to soft iron distortions, caused by deflections/alterations in the earth’s magnetic field and hard iron distortions, caused by objects that produce a magnetic field, magnetometers over periods of deployment are prone to accumulating position errors [28, 51, 52]. This is of particular consideration for diving species that spend extended periods underwater, where the impact of ocean currents may add to the degree of drift accumulated in the accelerometer/magnetometer sensors [53, 54]. Having quality, temporally regular locations, allows for successive correction of the position errors that can accumulate in dead-reckoning, in addition, accounting for the effects of current on dead-reckoning can lead to more accurate reconstructions of movement [53, 54].

This method provides an example of analysing space-use that could be integrated for a range of benthic foragers. Dead-reckoning and the collection of accelerometer/magnetometer data provides particular utility for species that spend extended periods underwater and surface briefly [55, 56], incurring large intervals between collected GPS or Argos locations. Additionally, the lower power consumption of accelerometers/magnetometers is especially useful for supporting extended tracking of both marine and terrestrial species, where the power requirement on archival and satellite-linked GPS systems is higher due to the duration of deployment [16, 22]. Accelerometer/magnetometer data can hence allow for an increased longevity in GPS devices throughout extended deployments, as sampling/transmitting frequencies of GPS systems can be lowered and movements between locations can be reconstructed.

While this method involves considerable, multiple-stage analyses for different data streams, the supplied code offers detailed step-by step instructions (in addition to vignettes for the referenced software packages) that allow easy adaptation of this method for different target species. In future, this method could be augmented significantly if used in conjunction with concurrent habitat/prey data. Furthermore, for a larger sample size, this method could be adapted to identify key foraging areas, at for example, a population or colony level. This would provide a powerful spatial tool for analysing habitat and resource-use for both marine and terrestrial species. For Australian sea lions for example, if this method was united with habitat and prey data collected via animal-borne video, this would allow important benthic habitat features and key prey-exploitation to be identified.

## Conclusions

In this study, we have developed a novel method for identifying key areas for a benthic-foraging pinniped. Our research has illustrated distinct spatial partitioning of benthic foraging effort by Australian sea lions and individual differences in space-use. It has also revealed tortuous foraging movements used by Australian sea lions, highlighting fine-scale use of key benthic habitats. This has shown the utility of tri-axial magnetometry/accelerometry in illustrating the sub-surface movements of a diving, marine species at high resolution. This provides additional information that would otherwise be missed by location and dive data alone, or more traditional models that predict straight-line movement between surface locations. More broadly, this shows the continuing evolution of magnetometry and accelerometry as powerful tools for highlighting key areas for species, beyond solely identifying shifts in animal movement.

The utility of this method; combining dead-reckoning and cluster analysis, is not restricted to benthic-foraging, marine species and could be adapted to a range of marine and terrestrial species. Combining this method with concurrent habitat and prey data, for example from remote sensing or animal-borne video would further increase its power as a tool for spatial analysis. This would allow the identification and mapping of important habitats and areas of core prey-utilisation. For species that require targeted management and conservation, an intimate understanding of their space-use is crucial to ensuring effective efforts and outcomes for populations.

## Electronic supplementary material

Below is the link to the electronic supplementary material.

Supplementary Material 1

## Data Availability

The datasets used and/or analysed during the current study are available from the corresponding author on reasonable request. The developed *R* code is freely available via the corresponding depository.

## Abbreviations

*GPS*: Global Positioning System
*Hz*: Hertz
*IUCN*: International Union for the Conservation of Nature
*TDR*: Time-depth recorder
*µT*: Micro tesla
*VHF*: Very high frequency
