# Supplementary material for: A novel method for identifying fine-scale bottom-use in a benthic-foraging pinniped
Source: Mov Ecol. 2023 Jun 9;11:34. doi: 10.1186/s40462-023-00386-1 (PMC10257308; doi:10.1186/s40462-023-00386-1)

***Additional Information 1***

**Supplementary Table 1.** Age, body metric, condition and reproductive data for three microchipped adult female Australian sea lions (at deployment) from Seal Bay Conservation Park

| **ID** | **Age (years)** | **Length (cm)** | **Girth (cm)** | **Mass (kg)** | **Lactating** | **Body condition** | **Pup age** | **Moult stage** |
| --- | --- | --- | --- | --- | --- | --- | --- | --- |
| **SB1** | 14.1 | 160 | 113 | 92.9 | Yes | Excellent | 8 months | Nearly all moulted |
| **SB2** | 5.4 | 152 | 102 | 75.8 | Yes | Good | 13 months | Post-moult brown |
| **SB3** | 17.4 | 160 | 112 | 85.2 | Yes | Very good | Juvenile, 2.5 years | Post-moult grey/fawn |

**Supplementary Figure 1.** Average silhouette width plots to identify the optimal number of clusters for partitioning of bottom-use for three adult female Australian sea lions. Individuals SB1, SB2 and SB3 are shown in plots a, b and c respectively. Average silhouette width scores are included in bold.


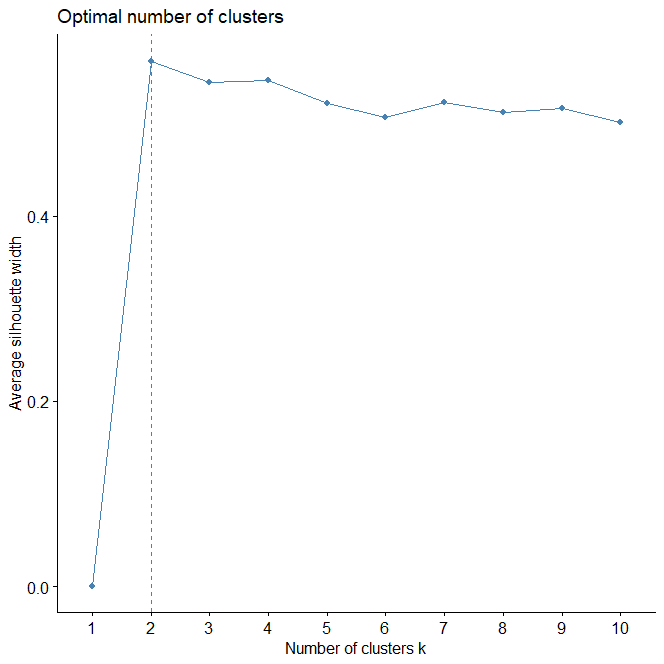


**a**

**SB1**

**0.57**


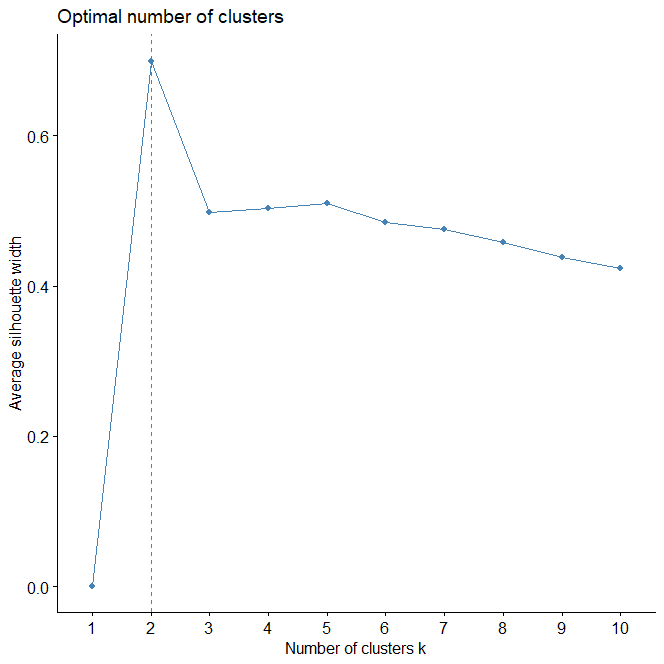


**b**

**SB2**

**0.66**


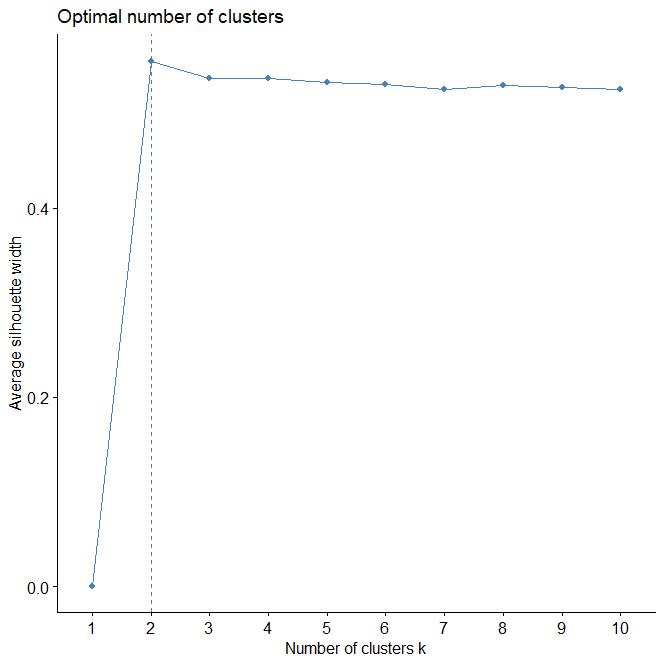


**c**

**SB3**

**0.56**

**Supplementary Figure 2.** Two-dimensional dive profile from the foraging path of an adult female Australian sea lion (SB1) from Seal Bay, highlighting their ‘U’ shaped benthic foraging dives at depth (metres). Dive profile is highlighted in blue for ten consecutive foraging dives.


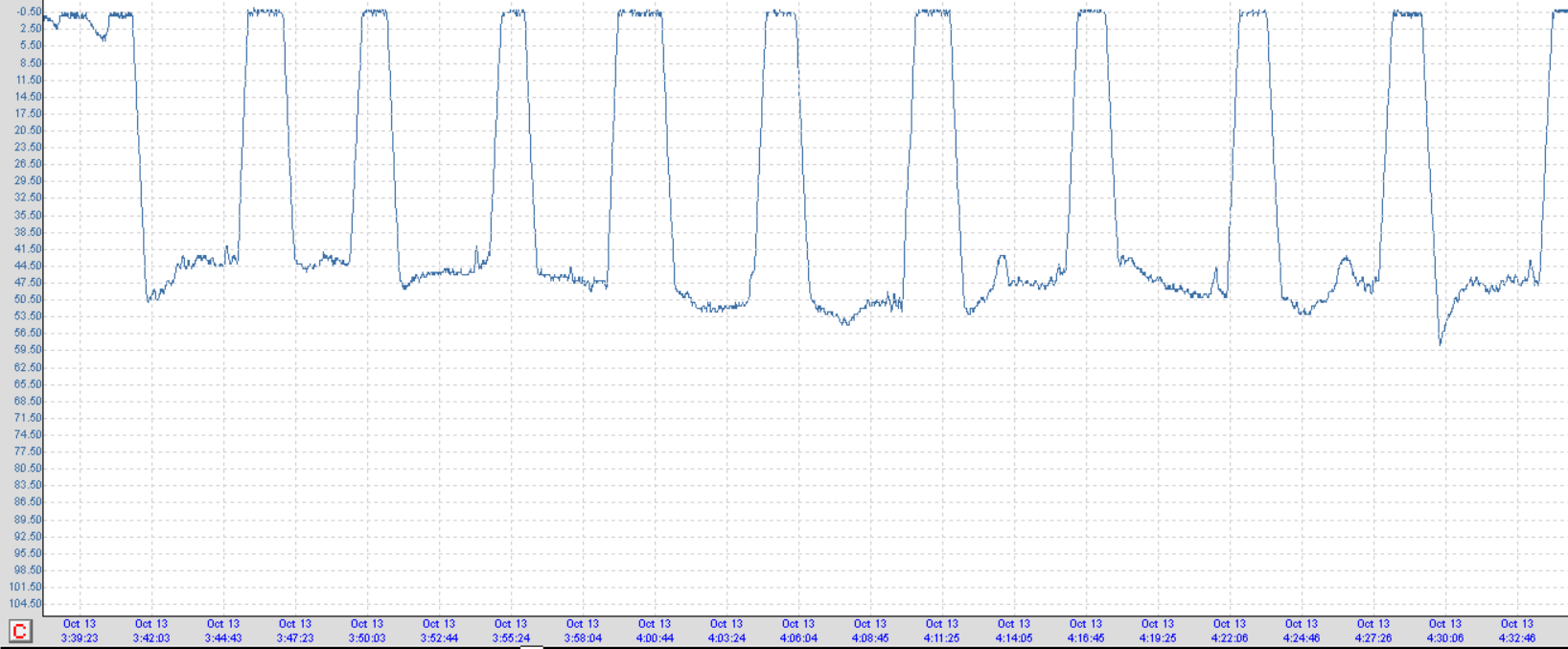

Supplement: Supplementary file 1 — Supplementary Material 1 [file 40462_2023_386_MOESM1_ESM.docx]
